# Which Treatment for Postherpetic Neuralgia?

**DOI:** 10.1371/journal.pmed.0020238

**Published:** 2005-07-26

Postherpetic neuralgia (PHN) is a chronically painful condition that is a complication of shingles (acute herpes zoster), a recurrence of the varicella-zoster virus, which initially causes chickenpox. Although shingles usually resolves within a month, some people continue to feel the pain of PHN long after the rash and blisters heal, because of nerve damage (neuropathic pain) caused by the shingles. Not everyone who has had shingles develops PHN, although it is a common complication of shingles in older adults.

Despite advances in antiviral therapy during acute herpes zoster and the more recent introduction of vaccination against varicella zoster, PHN continues to be a significant clinical problem, with 10–20% of patients developing persistent neuropathic pain after acute herpes zoster reactivation. The nature of PHN pain is variable, which implies that a variety of mechanisms might be operating. This variability has led to the hypothesis that treatment plans could be optimised for individual patients on the basis of the individual pattern of their symptoms or the underlying mechanism of the pain.

However, the current evidence base for therapies in PHN is based on clinical trials of analgesics, which have examined PHN as a single disease entity. Furthermore, there is little evidence for the efficacy of drugs for specific sets of symptoms and no simple way to determine which pain mechanisms might be operating in an individual patient.

In this month's *PLoS Medicine*, Andrew Rice and colleagues reassessed the evidence base by doing a systematic review and meta-analysis of analgesic therapy for PHN, which has fundamentally changed in the wake of several major new trials. The authors searched the literature for trials of PHN and retrieved 62 articles, of which 35 were kept for final analysis.

Their analysis confirmed several previous research findings, although they cautioned that the meta-analytic study design of collecting data from a range of trials had several inherent pitfalls, and it is difficult to directly compare treatments across different trials.

However, they found evidence for analgesic efficacy in established PHN for orally administered therapies, such as tricyclic antidepressants, some opioids, gabapentin, tramadol, and pregabalin. Some topically administered therapies, such as lidocaine and capsaicin, were associated with analgesic efficacy in selected patients. However, it appeared that therapies such as oral administration of certain NMDA receptor antagonists, codeine, ibuprofen, lorazepam, 5HT1 receptor agonists, and acyclovir were not efficacious in PHN.

Altogether, the authors conclude that the evidence base supports the first-line use of a tricyclic antidepressant for orally administered treatment of PHN, reserving the gabapentinoids for second-line use. Topical treatments, such as lidocaine or capsaicin, should be considered as first-line treatment if a patient falls into the “sensitised nociceptor” as opposed to “deafferentation” sub-group of PHN patients.

The role of intrathecal steroid is still not clear: one trial indicated that intrathecal steroids were associated with benefits in patients with PHN, but this therapy might be hazardous, and the authors and other researchers have concluded that further high-quality trials of this therapy are needed. The authors found little evidence regarding possible synergistic effects of the various treatments to support or refute the concomitant use of combinations of drugs.

They stressed that any treatment plan must recognise the importance of the biopsychosocial model of chronic pain and that any pharmacologically based management of PHN should be combined with advice on and management of psychological and social aspects.

Finally, as there is no single pathophysiology that underlies PHN, they propose that future studies should use quantitative sensory evaluation to clearly categorise subsets of participants for better interpretation of treatment effects.

